# Choosing between GLP-1 Receptor Agonists and DPP-4 Inhibitors: A Pharmacological Perspective

**DOI:** 10.1155/2012/381713

**Published:** 2012-10-18

**Authors:** Dominique Xavier Brown, Marc Evans

**Affiliations:** Department of Diabetes, University Hospital Llandough, Cardiff CF64 2XX, UK

## Abstract

In recent years the incretin therapies have provided a new treatment option for patients with type 2 diabetes mellitus (T2DM). The incretin therapies focus on the increasing levels of the two incretin hormones, glucagon-like peptide 1 (GLP-1) and glucose-dependent insulinotropic polypeptide (GIP). This results in increased glucose dependent insulin synthesis and release. GLP-1 receptor agonists such as liraglutide and exenatide exert an intrinsic biological effect on GLP-1 receptors directly stimulating the release of insulin from pancreatic beta cells. DPP-4 inhibitors such as sitagliptin and linagliptin prevent the inactivation of endogenous GLP-1 and GIP through competitive inhibition of the DPP-4 enzyme. Both incretin therapies have good safety and tolerability profiles and interact minimally with a number of medications commonly prescribed in T2DM. This paper focuses on the pharmacological basis by which the incretin therapies function and how this knowledge can inform and benefit clinical decisions. Each individual incretin agent has benefits and pitfalls relating to aspects such as glycaemic and nonglycaemic efficacy, safety and tolerability, ease of administration, and cost. Overall, a personalized medicine approach has been found to be favourable, tailoring the incretin agent to benefit and suit patient's needs such as renal impairment (RI) or hepatic impairment (HI).

## 1. Introduction

The pathophysiology of type 2 diabetes mellitus (T2DM) is complex and involves many facets. Currently a combination of metformin and lifestyle alterations is the intervention of choice. However, due to the progressive nature of T2DM, inevitably other supplementary therapies are often needed [1]. This has led to the development and approval of the incretin-based therapies targeting the incretin system, dysregulation of which arguably plays an important role in the pathogenesis of T2DM. The incretin system can briefly be summarised as the amplification of insulin biosynthesis and secretion due to the actions of two key hormones, glucagon like peptide 1 (GLP-1) and glucose dependent insulinotropic polypeptide (GIP) [2, 3]. GLP-1 and GIP are collectively known as the incretin hormones and are primarily released from the gastrointestinal tract upon ingestion of oral glucose substance [4]. In healthy individuals the insulin response to oral glucose is therefore much higher than to IV glucose, illustrating the potentiating effect of the incretin hormones. In patients with T2DM, the insulin response to oral glucose is similar to IV glucose, providing evidence that the incretin response is lost in these individuals. Modulation of the incretin system is therefore a viable treatment option and has had reasonable success in the form of two currently approved therapies, dipeptidyl peptidase 4 (DPP-4) inhibitors and GLP-1 receptor agonists [5]. With these new treatment options come new possibilities and options for clinicians but questions still remain, where do these new incretin therapies fit in with clinical practice and when should each therapy be prescribed? This paper aims to assess the benefits and pitfalls of each therapy from a pharmacology perspective.

## 2. Pharmacology of GLP-1 Receptor Agonists & DPP-4 Inhibitors

As mentioned above, the incretin hormones consist of GLP-1 and GIP, both released upon the ingestion of oral glucose substance. The relative importance of GLP-1 and GIP has been hotly debated. However, in T2DM the insulinotropic activity of GIP is negligible in contrast to GLP-1 [6]. Most attempts to modulate the incretin system are therefore directed at GLP-1. GLP-1 is a 30 amino acid peptide hormone, secreted by L cells of the terminal ileum in response to glucose, amino acids, and fats [7]. GLP-1 stimulates glucose dependent insulin release from pancreatic beta cells and suppresses glucagon release [5]. Exogenous administration of GLP-1 has been shown to be effective in restoring the first phase insulin response. A study in 2002 by Zander and colleagues also demonstrated that patients with T2DM administered GLP-1 exhibited decreased fasting plasma glucose (FPG) and postprandial glucose (PPG) levels [8]. However, GLP-1 has a circulating half-life of only ~1.5 mins as it is inactivated rapidly by the DPP-4 enzyme [9]. This has led to two different approaches to boosting the circulating levels of the incretin hormones. The first is distinctly pharmacological and involves creating GLP-1 mimetics which are more resistant to inactivation by DPP-4. These GLP-1 mimetics are agonists at the GLP-1 receptor and exert intrinsic biological activity, directly stimulating the release of insulin from pancreatic beta cells [10]. The second approach involves inhibiting the DPP-4 enzyme resulting in increased physiological levels of the incretin hormones GLP-1 and GIP [5].

Currently GLP-1 agonists have a higher status in the second line treatment of T2DM as stated in the guidelines from the American Diabetes Association [11] and the American Association of Clinical Endocrinologists [12]. Two GLP-1 receptor agonists exenatide and liraglutide are currently licensed in the USA, Europe, and Japan [13], however many more are in development. Exenatide is an exendin-4 GLP-1 mimetic with ~53% homology to endogenous GLP-1, it is currently approved as a monotherapy or in combination with metformin and/or sulphonylureas [14]. Liraglutide in contrast is a GLP-1 analogue with ~97% homology to human endogenous GLP-1. The 3% difference in homology results from the addition of a C16 fatty acid side chain, prolonging half-life and enabling liraglutide to be administered as a once daily dose [13]. Liraglutide and exenatide are administered subcutaneously (SC) via pre-filled injection pens once and twice daily, respectively. An additional exenatide once weekly preparation was approved in February 2012 by the US Food and Drug Administration (FDA). As these GLP-1 receptor agonists are able to be administered at supraphysiological doses this results in high circulating levels of GLP-1. At a pharmacological level this elicits a greater insulin response than DPP-4 inhibitors, which function only within physiological parameters [10].

The current DPP-4 inhibitors available are saxagliptin, sitagliptin, and linagliptin licensed in most of the world, additionally vildagliptin is licensed in Europe and Latin America, and alogliptin is licensed only in Japan [15–17]. DPP-4 inhibitors are competitive inhibitors of the DPP-4 transmembrane glycoprotein resulting in increased physiological levels of the incretin hormones [18]. DPP-4 inhibitors are considered second line treatments for T2DM either as a monotherapy or in combination with metformin and/or sulphonylureas [19]. One advantage of DPP-4 inhibitors lies in the fact that they are administered orally, increasing compliance. Most of the DPP-4 inhibitors available do not interfere with the cytochrome P450 (CYP) pathway with the exception of saxagliptin, metabolised by CYP 3A4 [20]. It is noteworthy that linagliptin is particularly different to the other drugs of its class as it is not extensively renally excreted and eliminated primarily via the hepatic route. This is an important development and will be discussed, as suitability for renal impairment (RI) is a desirable characteristic in any antidiabetic drug [21].

## 3. Prescribing Considerations

Several factors are to be considered when selecting between DPP-4 inhibitors and GLP-1 agonists for the second line treatment of patients with T2DM. These include but are not limited to glycaemic efficacy in terms of the ability to reduce haemoglobin A1c (HbA1c), FPG, and PPG levels; nonglycaemic efficacy; mechanistic considerations; effects on pancreatic beta-cell function; safety and tolerability; ease of administration; cost [1].

## 4. Glycaemic Efficacy: Clinical Studies

The results of several phase 2 and 3, double-blind, randomized controlled, or active comparator trials have shown that GLP-1 receptor agonists generally produce greater reductions in HbA1c and blood glucose than DPP-4 inhibitors. GLP-1 receptor agonists also promote significantly more weight loss than DPP-4 inhibitors which are weight neutral. These observations have been made in most cases indirectly through systematic reviews and meta-analyses of incretin studies as very little data is available from direct comparisons [1, 10]. This section provides an overview of the limited data available on glycaemic efficacy from selected head-to-head incretin studies. Most studies relate to comparisons made between GLP-1 receptor agonists and the DPP-4 inhibitor sitagliptin, therefore only extrapolation can be made regarding other DPP-4 inhibitors.

### 4.1. Exenatide versus Liraglutide

A 26-week randomized, open-label, multinational, parallel group study by Buse and colleagues compared the effectiveness of exenatide with liraglutide, both GLP-1 receptor agonists [22]. A total of 389 subjects completed the initial study with 202 randomized to once daily liraglutide and 187 randomized to twice daily exenatide. Subjects were adults with inadequately controlled T2DM currently taking maximally tolerated doses of metformin, sulphonylurea, or both. Liraglutide was administered as a once daily 1.8 mg dose and exenatide as a twice daily 10 *μ*g dose. The primary outcome measured was change in HbA1c from baseline. Liraglutide was found to decrease HbA1c levels significantly more than exenatide, −1.12% versus −0.79%, respectively (*P* < 0.0001). In terms of clinical significance this resulted in a greater number of subjects achieving the target HbA1c of <7% (*P* = 0.0015). Liraglutide also achieved a greater reduction in FPG than exenatide, −1.61 mmol/L versus −0.6 mmol/L, respectively (*P* < 0.0001). Both drugs were tolerated well by subjects and resulted in similar weight losses, −3.2 kg versus −2.87 kg, respectively. Liraglutide produced less nausea and minor hypoglycaemic episodes than exenatide.

### 4.2. Once Weekly Exenatide versus Twice Daily Exenatide

GLP-1 receptor agonists are administered by SC injection, making them less convenient and potentially reducing compliance. Drucker and colleagues conducted a 30-week, randomized study comparing the efficacy of a long acting 2 mg once weekly exenatide formulation with the traditional 10 *μ*g twice daily exenatide [23]. Subjects recruited were suffering from T2DM and were drug naive or currently taking one or more antidiabetic agents. Primary outcome was a change in HbA1c. Subjects administered exenatide once weekly displayed significantly greater reductions in HbA1c than those administered exenatide twice daily, −1.9% versus −1.5%, respectively (*P* = 0.0023). In terms of clinical significance, this resulted in significantly more patients achieving the target HbA1c of <7% (*P* = 0.0039). This study showed the more convenient once weekly formulation of exenatide actually displayed greater reductions in HbA1c than twice daily exenatide making it an important option in the treatment of T2DM. However, exenatide once weekly is slightly more complicated to prepare and administer than twice daily exenatide [24].

### 4.3. Exenatide versus Sitagliptin

DeFronzo and colleagues conducted a double-blind, randomized, crossover, multicentre study into the effectiveness of exenatide versus sitagliptin in subjects with T2DM currently on metformin therapy [25]. Subjects were administered exenatide 5 *μ*g twice daily for 1 week followed by 10 *μ*g twice daily for 1 week. Following this, subjects crossed over to the alternate therapy. The results demonstrated that 2-hour PPG was significantly reduced in subjects receiving exenatide versus sitagliptin 133 mg/dL versus 208 mg/dL, respectively (*P* < 0.0001). During the crossover period, switching to sitagliptin therapy from exenatide resulted in an increase of 73 mg/dL in 2-hour PPG levels. Similarly switching to exenatide from sitagliptin resulted in a 76 mg/dL decrease in 2-hour PPG levels. Exenatide and sitagliptin exerted similar reductions on FPG, −15 mg/dL versus −19 mg/dL, respectively. However, exenatide had a markedly greater effect on the insulinogenic index (*P* = 0.0239) and decreased postprandial glucagon secretion significantly more than sitagliptin (*P* = 0.0011). Exenatide also reduced total caloric intake to a greater extent than sitagliptin, −134 kcal versus +130 kcal. DeFronzo and colleagues concluded that although the study was short with a duration of only 2 weeks, the significant advantages displayed by exenatide had important clinical implications.

Bergenstal and colleagues conducted a multicentre 26-week, double-blind, double-dummy study in individuals with inadequately controlled T2DM despite current metformin therapy [26]. Subjects were stratified into cohorts and received either once weekly 2 mg exenatide injection plus daily oral placebo, or once weekly placebo injection plus daily 100 mg oral sitagliptin, or once weekly placebo injection plus daily 45 mg oral pioglitazone therapy. The primary outcome measured was a change in HbA1c levels from baseline. Subjects treated with exenatide exhibited greater reductions in HbA1c than those treated with sitagliptin, −1.5% versus −0.9% respectively (*P* < 0.0001). Weight loss with exenatide was also significantly more than that with sitagliptin, −2.3 kg versus −1.5 kg, respectively (*P* = 0.0002). This confirms previous studies suggesting that GLP-1 receptor agonists are more potent agents in glycaemic control.

### 4.4. Liraglutide versus Sitagliptin

A multicentre, open-label, parallel group study by Pratley and colleagues compared the effectiveness of liraglutide with sitagliptin in subjects with inadequately controlled T2DM despite current metformin therapy [27]. Subjects were randomized to either once daily 1.2 mg or 1.8 mg liraglutide, or 100 mg oral sitagliptin once daily. The primary outcome was a change in HbA1c from baseline. Liraglutide 1.8 mg and 1.2 mg displayed superiority in glycaemic control as expected compared to sitagliptin, −1.5%, −1.24%, and −0.9%, respectively (*P* < 0.0001). Hypoglycaemia was uncommon in all treatment groups (5%). Nausea was, however, more common with liraglutide 1.8 mg (27%) and 1.2 mg (21%) than with sitagliptin 100 mg (5%). The study concluded that liraglutide was more effective than sitagliptin in terms of glycaemic control and was well tolerated; however, the very small number of adverse events experienced by subjects receiving sitagliptin demonstrates their potential place in clinical practice.

### 4.5. Sitagliptin versus Saxagliptin

The results of this trial have been included to give an indication of sitagliptins efficacy in relation to other DPP-4 inhibitors. Scheen and colleagues carried out an 18-week, double-blind, randomized, multicentre, noninferiority trial to assess the effectiveness of sitagliptin and saxagliptin as add-on therapies to metformin. Subjects recruited were suffering from inadequately controlled T2DM despite current metformin therapy. Subjects received daily doses of 5 mg saxagliptin or 100 mg sitagliptin in addition to metformin. The primary outcome was a change in HbA1c from baseline. The results demonstrated that saxagliptin and sitagliptin both reduced HbA1c by −0.52% and −0.62%, respectively. As this trial was designed to assess noninferiority the results cannot be used to assess which DPP-4 inhibitor is more effective. However, it seems clear that saxagliptin is not inferior to sitagliptin in terms of glycaemic control and indicates that study data can be extrapolated to some degree to other DPP-4 inhibitors.

## 5. Nonglycaemic Efficacy

### 5.1. Weight

Strong evidence suggests an association between increased weight and the incidence of T2DM and an increase in morbidity and mortality in patients with T2DM. A systematic review by Ross and colleagues concluded that weight management should be an essential factor when selecting an appropriate antidiabetic therapy, with even a 1 kg weight loss being significantly beneficial [28]. GLP-1 receptor agonists have demonstrated their ability to produce significant weight losses in several systematic reviews and meta-analyses [29, 30], producing weight losses of between −2.8 kg to −4.8 kg [31]. In contrast, DPP-4 inhibitors are weight neutral [32] as described in several studies compared to placebo treatment. These differences suggest that GLP-1 receptor agonists may provide many patients the ability to lose and maintain weight effectively whilst achieving adequate glycaemic control [31]. This difference between GLP-1 receptor agonists and DPP-4 therapies may stem from a pharmacological perspective. Endogenous GLP-1 acts to reduce gastric emptying and inhibit upper intestinal motility. This results in increased satiety and decreased food intake. Although both incretin therapies cause increased levels of GLP-1, DPP-4 inhibitors only achieve this within physiological parameters. GLP-1 receptor agonists on the other hand are administered at pharmacological doses potentially resulting in far increased, longer lasting effects on satiety.

### 5.2. Pancreatic Beta-Cell Pathogenesis

The pathogenesis of T2DM results in a decreased pancreatic beta-cell mass, progressively damaging the first phase insulin response. If glycaemic control cannot be achieved through first and second line antidiabetic agents, insulin is the final agent of choice; however, insulin therapy comes with significant risks of adverse events such as hypoglycaemia. Therefore, any antidiabetic agent which improves beta-cell function is preferable. Several studies described below have linked this loss of beta-cell mass and function to decreased levels of the incretin hormones [33].

Farilla and colleagues conducted an *in vitro* study using isolated human pancreatic islets to ascertain the effects of GLP-1 on beta-cell mass and neogenesis. The human islets were cultured for 5 days with GLP-1 or a control followed by an analysis of general viability, proapoptotic and anti-apoptotic markers. Three-dimensional morphology of the pancreatic islets was improved in the GLP-1-treated pancreatic islets compared to control islets. Nuclear condensation was also decreased in pancreatic islets treated with GLP-1 with a decrease in the apoptotic enzyme caspase 3 compared to controls (*P* < 0.001). The study concluded that GLP-1 improves beta-cell morphology whilst improving function and inhibiting apoptosis [34].

This study by Farilla and colleagues sparked interest into the mechanism of action by which GLP-1 inhibits apoptosis in pancreatic beta cells and improves glucose responsiveness. Extensive analysis of the *in vitro* and *in vivo* evidence for the mechanism of action of GLP-1 in improving beta cell function is beyond the scope of this paper. However, Song and colleagues postulate that the majority of the effects of GLP-1 on beta-cells are exerted through intracellular signalling pathways involving cyclic adenosine monophosphate (cAMP) and protein kinase A (PKA) [35]. This pathway is activated through the binding of GLP-1 to the G-protein coupled GLP-1 receptor, leading to pleiotropic effects such as beta-cell neogenesis and decreased apoptosis.

An *in vitro* study conducted by Cunha and colleagues demonstrated the protective effects of GLP-1 agonists on rat beta cells [36]. GLP-1 agonists were found to exert a protective effect on the beta cells when exposed to free fatty acids (FFAs) via the induction of the endoplasmic reticulum (ER) chaperone BiP and the antiapoptotic protein JunB. Similarly the DPP-4 inhibitor vildagliptin has been shown to improve beta cell function in rats *in vivo*. In this study vildagliptin was administered orally to neonatal rats over a period of 5 to 19 days. Following this, the pancreatic beta cells were extracted and displayed an increase in mass and reduction in apoptotic markers [37]. Although these studies by Farilla, Song, and Duttaroy show a clear link between the incretin hormones and beta cell function, there is very little evidence available for how this translates in humans. Although there is some data indicating that beta cell morphology and function is improved in humans, it is not feasible to extrapolate this to clinical practice until further long-term data in humans is available.

## 6. Economics

The cost of antidiabetic agents is an important global factor to be considered when prescribing. This can be difficult and depends on the definition of cost. For example, as T2DM is a condition with significant multisystemic effects, total diabetes-related costs may differ from total medical costs. In terms of pure cost, DPP-4 inhibitors are less expensive than GLP-1 receptor agonists. However, as discussed by Scheen and colleagues, the dose of the drug and mode of administration can cause variations in the overall cost, such as 1.2 mg versus 1.8 mg liraglutide and once weekly exenatide versus twice daily exenatide [38]. A health economic analysis into the cost effectiveness of exenatide and sitagliptin found that these new incretin therapies may incur a significant cost to healthcare systems. Sitagliptin and exenatide had an incremental cost-effectiveness ratio of $169,572 and $278,935, respectively, per quality-adjusted life years (QALY) gained [39]. This would appear to suggest that sitagliptin offers superior cost effectiveness; indeed a similar result from an independent study demonstrated that diabetes-related medical costs were lower with sitagliptin compared to exenatide. However, in the same study exenatide offered lower 6-month total medical costs despite some diabetes-related components being more expensive [40]. These studies put into perspective the difficulties in assessing the cost effectiveness of GLP-1-and DPP-4-based therapies, perhaps more long-term data will elucidate the true cost benefit of these agents.

## 7. Renal Impairment

Chronic kidney disease (CKD) is a common complication of T2DM resulting in a progressive deterioration of renal function. CKD can be defined as being kidney damage or decreased kidney function for ≥3 months [41], it is characterised by 5 stages dependent on estimated glomerular filtration rate (eGFR). Achieving glycaemic control (HbA1c < 7%) is paramount in these patients in order to limit any further damage. However, many antidiabetic agents are contraindicated or require dosage adjustments dependent on the degree of renal dysfunction [42]. The pharmacokinetics of individual incretin therapies differ greatly even between drugs of the same class and largely dictate their suitability for patients with RI.

Exenatide and liraglutide have very different pharmacokinetic profiles with exenatide eliminated primarily via the kidneys and liraglutide undergoing generalized proteolysis. Importantly, both drugs appear to exhibit limited drug interactions with other agents [43]. Studies in pigs have shown that exenatide is solely cleared by glomerular filtration [44]; an open-label study was conducted in 31 patients with different degrees of RI to assess the effects on the pharmacokinetic profile of exenatide [45]. RI was classified by creatinine clearance (CrCL) with groups stratified into no RI (CrCL > 80 mL/min), mild RI (CrCL 51–80 mL/min), moderate RI (CrCL 31–50 mL/min), or end-stage renal disease (ESRD). The half-life of twice daily 5 *μ*g and 10 *μ*g exenatide in healthy, mild, moderate RI and ESRD patients was 1.5 h, 2.1 h, 3.2 h, and 6 h, respectively. Exenatide appeared to be well tolerated in mild and moderate RI cohorts; however, was not well tolerated by patients with ESRD with adverse events of nausea and vomiting. Although the study had a small sample size it demonstrated that the effect on the pharmacokinetic profile of exenatide was clinically acceptable in the mild and moderate RI cohorts; however, at the current available doses it was not suitable for patients with ESRD. Notable to mention is also the effect of RI on the pharmacokinetic profile of once weekly exenatide, recently approved by the FDA in the USA and added to National Institute for Clinical Excellence (NICE) guidelines in the UK. Mild and moderate RIs were found to increase systemic exposure of exenatide once weekly by 23% and 74%. Exenatide once weekly is therefore only suitable for patients with mild RI and contraindicated in patients with moderate RI and above [24].

As described above, liraglutide is not eliminated via the kidneys but undergoes a more generalized proteolysis. A study involving 7 healthy males administered radio-labelled liraglutide revealed that the drug is cleaved by DPP-4 much like endogenous GLP-1, albeit at a much slower rate. No intact liraglutide is excreted indicating that it is fully metabolized [43, 46]. Clinical studies have shown that renal or hepatic impairment does not alter the pharmacokinetic profile of liraglutide significantly. One study involved administering 0.75 mg SC liraglutide to 30 subjects with varying degrees of RI [47]. Area under the curve (AUC) did not demonstrate equivalence between patients with severe RI and healthy renal function. However, a regression analysis of log (AUC) showed that decreased CrCL did not appear to affect the pharmacokinetic profile of liraglutide significantly. Varying degrees of RI did not appear to be associated with incidence of adverse events. The study concluded that liraglutide can be used safely in patients with varying degrees of RI and even ESRD as this did not affect exposure. However the long-term data regarding the use of liraglutide in patients with moderate-to-severe RI is sparse leading to the summary product characteristics (SPCs) of liraglutide not recommending the use of liraglutide in moderate to severe RI [48].

The DPP-4 inhibitors display significant differences in their chemical structure, leading to different structure activity relationships (SARs) and variations in pharmacokinetic profiles [43]. Sitagliptin is an important DPP-4 inhibitor primarily excreted renally. An open-label study in 30 subjects with varying degrees of RI was conducted to assess the effects on the pharmacokinetic profile of sitagliptin [49]. All subjects received a 50 mg oral dose of sitagliptin. AUC was significantly increased in subjects with mild, moderate, severe RI and ESRD compared to healthy subjects, 1.61-, 2.26-, 3.77- and 4.50-fold, respectively. Based on these results it is recommended to make dose adjustments in RI with the normal 100 mg daily dose reduced to 50 mg in moderate RI and 25 mg in severe RI and ESRD. As expected an open-label single dose study revealed that hepatic impairment (HI) did not significantly affect the pharmacokinetic profile of sitagliptin [50].

Saxagliptin is the only DPP-4 inhibitor to undergo significant hepatic metabolism and is subject to biotransformation by the cytochrome P450 (CYP) 3A4 isozyme. This results in the production of the active metabolite BMS-510849 [43]. HI is therefore a potential hinderence to the use of this agent and has been evaluated in an open-label 10 mg study in subjects with differing degrees of HI [51]. Unfavourable Child Pugh scores (measure of HI) were associated with decreased saxagliptin clearance. AUC of saxagliptin was increased from +10% to +77% with an increased severity of HI and the active metabolite BMS-510849 decreased from −7% to −33%. As BMS-510849 displays ~2-fold less inhibitory potency for DPP-4 than intact saxagliptin, this indicates that HI will have significant effects on the safety of saxagliptin. In view of the effects of HI on the pharmacokinetic profile of saxagliptin it is not recommended that saxagliptin is used in patients with severe HI. Drugs which are metabolised by CYP isozymes especially CYP 3A4 will undoubtedly have their pharmacokinetic profile affected or affect the profile of other drugs. The potential for drug interactions with saxagliptin is low; however, care must be taken when prescribing alongside inhibitors or inducers of CYP 3A4 such as diltiazem, rifampicin, and ketoconazole [43]. In terms of RI, an open-label 10 mg study was conducted to assess the effects on saxagliptins pharmacokinetic profile. The results demonstrated that severe RI increased the AUC of saxagliptin and BMS-510849 2.1, and 4.5-fold, respectively compared to healthy controls. A dose adjustment to 2.5 mg is therefore recommended in patients with moderate-to-severe RI [52].

Vildagliptin is a DPP-4 inhibitor licensed in Europe and Latin America. Two key clinical studies have established that vildagliptin is extensively metabolized preceding excretion through hydrolysis, glucuronidation, and oxidation [53, 54]. An *in vitro* study demonstrated that vildagliptin is hydrolysed in the liver with 23% excreted in unchanged form by the kidneys [43]. Accordingly, an open-label study in subjects with mild, moderate, and severe HI as well as normal hepatic function was conducted [55]. Subjects administered single oral doses of 100 mg vildagliptin. AUC and *C*_max⁡_ of vildagliptin were not found to be significantly different between subjects with any degree of HI. Although vildagliptin is extensively metabolized, as mentioned previously, ~23% vildagliptin is excreted unchanged in the urine, this brings into question the problem of RI. In the USA vildagliptin has not yet been approved due to the limited data available on RI and a potential for skin lesions in this patient group linked to increased exposure. In the EU, vildagliptin has recently received a license update and can be prescribed in patients with mild RI without dosage adjustment and in moderate-to-severe RI following appropriate dosage adjustments [56]. Unlike saxagliptin, vildagliptin is not metabolized extensively by the CYP enzymes and therefore it is not likely to have any significant drug-drug interactions with CYP inducers or inhibitors [43].

Linagliptin is a novel xanthene-based DPP-4 inhibitor unlike other drugs of the same class. No dose adjustments in RI are required with linagliptin. This has been demonstrated in a study involving 51 subjects with varying degrees of RI. The elimination and exposure of linagliptin were not significantly different between groups with mild-to-severe RI [57]. Linagliptin is arguably the most significant advancement in recent DDP-4-based incretin therapy as it can be prescribed in any degree of RI with no dose adjustments.

## 8. Cardiovascular Protective Effects

Beyond glycaemic control there are also important cardiovascular protective effects associated with the incretin therapies. Due to the distinctly pharmacological action of GLP-1 receptor agonists mentioned previously, they exert greater effects on lipid profile and blood pressure (BP) than DPP-4 inhibitors [27, 58, 59]. Reductions in systolic BP are modest with GLP-1 receptor agonists at ~1–7 mm Hg [10]. Studies with DPP-4 inhibitors do not indicate a significant effect on systolic BP [10, 26]. A 6-week, double-blind, randomized, multicentre, crossover study demonstrated that twice daily exenatide decreased triglyceride levels to a greater extent than sitagliptin (*P* = 0.0118) [25]. Similarly, a 26-week, double-blind study comparing liraglutide 1.2 mg and 1.8 mg to once daily 100 mg sitagliptin revealed that triglyceride levels were decreased by 17 mg/dL, 38 mg/dL, and 35 mg/dL, respectively. Total cholesterol was also reduced to a greater extent with liraglutide than sitagliptin (*P* = 0.0332) [27]. Further studies evaluating hard cardiovascular endpoints such as morbidity and mortality will elucidate which class of agents offers better cardiovascular protective effects.

## 9. Safety and Tolerability

Clinical studies have shown that both GLP-1 receptor agonists and DPP-4 inhibitors have good safety and tolerability profiles. All glucose-lowering agents have the potential to cause hypoglycaemia and a key attribute of the incretin therapies is their low risk of hypoglycaemia, which has been monitored closely in trials. Generally, it is accepted that DPP-4 inhibitors have an excellent and slightly better tolerability profile than GLP-1 receptor agonists with a lower incidence of adverse events such as gastrointestinal upset and nausea. These are relatively common adverse events associated with exenatide and liraglutide leading to dropouts in clinical studies [38]. This has been noted in clinical practice with more patients unable to tolerate GLP-1 receptor agonists than DPP-4 inhibitors. Common adverse events other than hypoglycaemia associated with GLP-1-and DPP-4-based therapies are listed in Table 1 [60].

The principal safety concerns of the incretin-based therapies at the current time are hypoglycaemia, nausea, acute pancreatitis, and hypersensitivity reactions which have presented in clinical studies and after the approval [60].

### 9.1. Hypoglycaemia

Close monitoring in trials has demonstrated the incretin therapies carry a low risk of hypoglycaemia. This is due to the pharmacological mechanism by which the incretin therapies function, stimulating only glucose dependent insulin release and limiting alpha-cell postprandial glucagon secretion [61]. In addition, glucagon suppression does not occur with the incretin therapies at plasma glucose levels <65 mg/dL, further reducing risk of hypoglycaemia [62]. In line with these mechanistic considerations, severe hypoglycaemia has not been reported in any clinical studies to date, except when incretin therapies are combined with other antidiabetic agents such as sulphonylureas [58, 60, 63]. Mild hypoglycaemia and moderate hypoglycaemia have however been reported in clinical studies of exenatide (4–9%) [58, 63] and liraglutide (0–12%) monotherapy [59]. Compared with sulphonylurea therapy such as glimepiride (24%) the incidence of hypoglycaemia is negligible [59]. As expected DPP-4 inhibitors such as sitagliptin (0–4%) display even lower incidence of mild-to-moderate hypoglycaemia with figures close to those achieved with placebo [60, 64]. Due to the low incidence of hypoglycaemia associated with the incretin-based therapies the American Diabetes Association recommends GLP-1 receptor agonists for groups of patients particularly prone to hypoglycaemic episodes and for those groups in which hypoglycaemia is particularly undesirable. This includes individuals who operate heavy and dangerous machinery and pilots [60].

### 9.2. Nausea

Nausea has presented as one of the most common adverse events associated with GLP-1 receptor agonists, noted with exenatide and liraglutide in several clinical trials [58, 63]. One particular head-to-head incretin study reported 28% of subjects experienced transient nausea with twice daily exenatide and 26% with once daily liraglutide [22]. This can be compared with DPP-4 inhibitors such as sitagliptin and saxagliptin which have nausea incidence rates of 1-2% and 2–4%, respectively, close to those achieved with placebo [10, 65, 66]. Although nausea presents as a more significant issue with GLP-1 receptor agonists, this can be allievated through dose escalation strategies. Exenatide twice daily can be prescribed at 5 *μ*g for the first 4 weeks, after this the dose can be escalated to 10 *μ*g if glycaemic control is not achieved [14]. Liraglutide can be prescribed at 0.6 mg daily for one week, followed by a dose escalation to 1.2 mg or 1.8 mg if glycaemic control is not achieved [48]. Importantly, patients must also be informed that nausea symptoms generally peak at ~8 weeks, as patient education regarding this issue will undoubtedly improve compliance.

### 9.3. Acute Pancreatitis

Acute pancreatitis has been confirmed as a potential adverse event associated with many of the incretin therapies such as exenatide [14], liraglutide [48], and sitagliptin [67]. This evidence has been gathered in clinical studies and after the approval. However, patients with T2DM are already at 2- to 3-fold increased risk of suffering from acute pancreatitis [68]. An analysis conducted of two insurance claims databases has demonstrated that the risk of pancreatitis was equivalent with most antidiabetic agents. One particular insurance analysis revealed that the incidence rates of pancreatitis per 1000 patient-years were 5.7, 5.6, and 5.6 for exenatide twice daily, sitagliptin, and metformin [69, 70]. Regardless, the FDA has requested that the manufacturers of the incretin therapies such as liraglutide, exenatide, sitagliptin, linagliptin, and saxagliptin conduct further epidemiological clinical studies into the incidence of acute pancreatitis [71]. The FDA has also recommended that patients at a particular risk of pancreatitis should avoid exenatide [14] whilst liraglutide [48] may be used with close monitoring. The DPP-4 inhibitors contain no safety information regarding prescribing to patients at risk of pancreatitis [19, 67]. Perhaps increasing patient education into how to spot the signs and symptoms of pancreatitis early will also help to reduce complications associated with this condition [10].

### 9.4. Hypersensitivity Reactions

Hypersensitivity reactions have been reported in clinical studies of liraglutide [48], twice daily exenatide [14], sitagliptin [67], linagliptin [19], and saxagliptin [52]. Some postmarketing surveillance systems have reported a small minority of serious hypersensitivity reactions with the GLP-1 receptor agonists exenatide [14]. Liraglutide has induced an immunogenic reaction in clinical studies with a small minority of patients (0.8%) experiencing urticaria, only 0.4% of patients experienced this with more traditional antidiabetic agents [48, 60]. The DPP-4 inhibitors such as sitagliptin can also induce hypersensitivity reactions. These reactions normally present within 3 months with sitagliptin which has been reported to cause anaphylaxis, angioedema, and exfoliative dermatitis in a minority of patients. With all incretin therapies, treatment should be immediately discontinued if a hypersensitivity reaction occurs [60].

## 10. Conclusion

The treatment of T2DM has become more complex in recent years with the addition of the incretin therapies as a new pharmacological option. It is clear that both GLP-1 receptor agonists and DPP-4 inhibitors have a valuable place in the second line treatment of T2DM. Their glycaemic efficacy is impressive although it cannot match the glucose lowering effects of metformin and sulphonylureas. GLP-1 receptor agonists appear to lower blood glucose to a greater extent and promote more weight loss than DPP-4 inhibitors, which are weight neutral. They also have increased cardiovascular protective effects with respect to improving lipid profile and systolic BP. However, a definite answer to which incretin therapy is most effective cannot be given until more long-term data is gathered from clinical trials assessing hard endpoints such as morbidity and mortality. The safety and tolerability profile of the incretin therapies is excellent and equal if not better in most cases than traditional therapies. However, DPP-4 inhibitors seem to have an edge over GLP-1 receptor agonists with an excellent tolerability profile. DPP-4 inhibitors also have the advantage of being administered orally whereas GLP-1 receptor agonists are administered by SC injection. This will also undoubtedly increase compliance in certain patient groups and potentially reduce the detrimental effects of hypoglycaemia in at-risk individuals. All in all, the question should not be whether one class of incretin therapy is superior, but instead whether individual incretin therapies are superior in particular patient groups. Personalizing incretin therapies to particular patients will allow effective glycaemic control whilst avoiding adverse events. This is particularly important in patients who suffer from HI or RI. For example, GLP-1 receptor agonists are not particularly suited to RI whereas the novel DPP-4 inhibitor linagliptin can be prescribed safely in any degree of RI. As in all medication choices there is usually a trade-off, in this case glycaemic efficacy is likely to be reduced compared to GLP-1 receptor agonists. Ultimately, long-term postmarketing surveillance and further clinical trials will elucidate the patient groups to which each incretin therapy is most suited.

## Figures and Tables

**Table 1:** Common adverse events associated with the incretin therapies other than hypoglycaemia: exenatide, liraglutide, sitagliptin, saxagliptin, and linagliptin, adapted from [60].

GLP-1 receptor agonists	Adverse events
Exenatide	Nausea, diarrhoea, vomiting, feeling jittery, dizziness, headache, and dyspepsia
Liraglutide	Nausea, diarrhoea, and headache

DPP-4 inhibitors	

Sitagliptin	Upper respiratory tract infection, nasopharyngitis, and headache
Saxagliptin	Upper respiratory tract infection, urinary tract infection, and headache
Linagliptin	Headache, joint pain, and sore throat
Linagliptin and metformin	Cough, decreased appetite, nausea, headache, sore throat, vomiting, and diarrhoea
